# Impact of haptic feedback on surgical training outcomes: A Randomised Controlled Trial of haptic versus non-haptic immersive virtual reality training

**DOI:** 10.1016/j.amsu.2022.104734

**Published:** 2022-09-23

**Authors:** Abrar Gani, Oliver Pickering, Caroline Ellis, Omar Sabri, Philip Pucher

**Affiliations:** a Department of Trauma and Orthopaedics, St George's University Hospitals NHS Foundation Trust, London, UK; b Department of General Surgery, Portsmouth University Hospitals NHS Trust, Portsmouth, UK

**Keywords:** Virtual reality (VR), Haptic feedback, Orthopaedic, Surgical simulation, Bone drilling, Training

## Abstract

**Objective:**

This study aimed to evaluate the educational impact of integrated haptic feedback in an immersive VR bone drilling simulation on the performance of a cohort of junior surgeons.

**Design:**

Block randomised, controlled, double-blinded study.

**Setting:**

St Georges University Hospital, London, United Kingdom.

**Participants:**

and methods: 31 trainee doctors (postgraduate years 1–3) with limited orthopaedic experience were recruited to participate in this randomised controlled study through e-mail and poster advertising. They were allocated to haptic or non-haptic group through block randomisation prior to entering the study environment. All participants provided verbal and written consent to participate in this study. All participants were blinded to the nature of the study as well as its intervention arms. All participants completed an immersive virtual reality training module with either haptic feedback or no haptic feedback in which they had to drill 3 bicortical holes in a VR tibia bone model in preparation for screw insertion followed by an ex vivo equivalent task on a tibial sawbone model once again drilling 3 holes through both cortices of the tibia. Outcome measures were plunge gap distance, drilling time and objective structures assessment of technical skills (OSAT) as well as qualitative questionnaire outcomes.

**Results:**

Haptic feedback in the VR training module showed significantly less plunge gap distance compared to the non-haptic group (7.6 mm ± 4.3 vs 13.6 mm ± 7.4 (p = 0.012)). The haptic group also had longer drill times (17.5 s ± 4.0 vs 13.8 s ± 4.2 (p = 0.027)), higher combined OSAT cores (14 (10,17) vs 8.5 (7.75, 12), p = 0.0006) and greater number of safe drills of <5 mm plunge gap in at least 2 out of 3 attempts (6 (40) vs 0 (0), p = 0.021.

**Conclusions:**

This study demonstrates better performance for an orthopaedic surgical task when using a VR-based simulation model incorporating haptic feedback, compared to one without haptic feedback supporting the pursuit and implementation of haptics in surgical training simulation models to enhance their educational value.

## Highlights

• Haptic Feedback in Virtual reality simulator increases performance of tibial bone drilling task compared with non-haptic virtual reality simulator.
• Virtual Reality with haptic feedback produces a safer drill in a bone drilling task with reduced risk of injury to neurovascular structures.
• Objective structured assessment of technical skills (OSATs) ratings was significantly improved in the haptic group.
• This study provides evidence that implementation of haptic feedback within surgical simulators will deliver a more effective and immersive experience for surgical trainees.

## 1 Introduction

The shift towards a competency-based system of progression, coupled with restricted clinical hours reducing trainees' operative exposure has placed a great emphasis on ‘ex vivo’ surgical training techniques [[1], [2], [3]]. Simulation models eliminate risks to patient safety during the very early stages of learning, facilitate skill acquisition, and improve clinical outcomes [4,5].

Advances in technology have allowed ever-increasing fidelity in simulations; immersive virtual reality (VR) simulation training represents the cutting edge in multisensory three-dimensional real-time interactivity. Entire procedures can be rehearsed, outcome metrics reflected upon, and performance improved through repetition in an immersive simulated clinical environment without compromise to patient safety. Previous VR training platforms have been shown to be effective in improving transferable operative skills and efficiency in a range of surgical specialities [[6], [7], [8]]. However, to date, most systems have been limited to audio and visual simulation, without the surgically crucial sense of touch or haptic feedback.

In surgery, haptics refers to the sense of touch and proprioception that a surgeon experience [9,10]. In open and endoscopic surgery, surgeons may use haptic feedback to discriminate tissue types, navigate dissection planes, and gauge the forces they are applying through their instruments to avoid tissue trauma [[10], [11], [12]]. Early studies of haptic feedback in VR simulation training have suggested benefits during the early phase of psychomotor skill acquisition [[13], [14], [15], [16]], but haptic surgical simulation technology has to date been nascent, with their true utility in training debated [17].

Existing studies assessing the impact of haptics in VR training to date are of poor quality, limited by significant bias risk and confounding factors [16]. Recently published small studies assessing haptics in VR bone drilling tasks have reported potential benefits, but have suffered from significant methodological limitations, small sample sizes (n < 10) and the lack of validated skills assessment [18,19].

Cortical bone drilling is a key skill in orthopaedic surgery wherein haptic feedback plays a key role to ensure the surgeon stops drilling just as the drill bit breaches the distal cortex of the bone. This results in a sudden release of resistance as the bone is penetrated, in order to prevent damage to adjacent soft tissue and vasculature.

This study aimed to evaluate the educational impact of integrated haptic feedback in an immersive VR bone drilling simulation on the performance of a cohort of junior surgeons in a double-blinded randomised controlled trial.

## 2 Methods

Following local institutional approval at St George's University NHS Trust, junior trainees (foundation year and core surgical trainees, representing non-specialised levels of training) were recruited across three urban London-area hospitals through e-mail and poster advertising to participate in this study hosted at St George's Hospital, London, United Kingdom. This randomised controlled trial was carried out in accordance with CONSORT criteria [20]. The study was also registered with the Research Registry (UIN: researchregistry8113) [21]. The recruitment period was for two weeks prior to the commencement of the study with the study period lasting a further two weeks.

Inclusion criteria were any trainees within the above specified training years, regardless of intended future specialty. Exclusion criteria were any trainees with prior experience with the VR training modules used in the study. In the absence of relevant pre-existing data, no sample size calculation was undertaken; a target sample size of 30 was set in order to exceed published sample sizes of comparable orthopaedic simulation studies [22].

Participants were block-randomised in daily 4–6 person blocks, using an online random number sequence generator, and allocated into either haptic or non-haptic study groups. Participants were aware only that they were taking part in a VR simulation training study and were blinded to the nature of the study, its intervention arms, and their group allocation. Hardware and any visual cues relating to the non-allocated group were stowed for each participant to prevent them from determining the nature of the other study arm.

A pre-procedure questionnaire and written consent to participate in the study was completed by participants. It assessed participants’ hand dominance, operative experience (observed, assisted, primary surgeon), prior experience with power drills, virtual reality, and video games.

### 2.1 Intervention

**Fig. 1:**
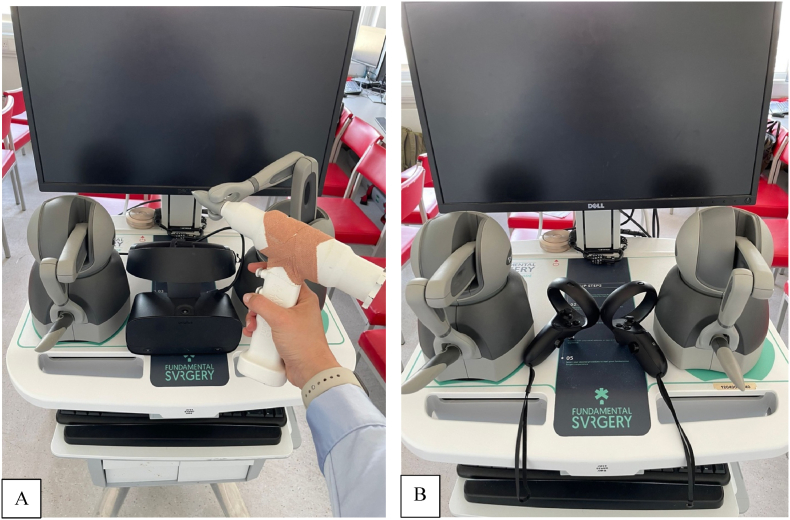
VR simulator setup for Drilling Study group A (Haptic) and group B (non-haptic).

Participants in both study groups completed one attempt of the immersive virtual reality training module immediately after completing the pre-procedure questionnaire. Participants used either an Oculus Rift (Meta Platforms, Cambridge, MA, USA) headset paired with GeoMagic (3D systems, Rock Hill, SC, USA) haptic feedback hand controllers (haptic group), or Oculus Rift headset and native Oculus Rift controllers (no haptic feedback, non-haptic group). (Fig. 1).

The training module was provided by an established surgical simulation company, (Fundamental Surgery, London, UK), with simulation modules and software identical for both groups with the exception of haptic feedback being disabled in the non-haptic group.

**Fig. 2:**
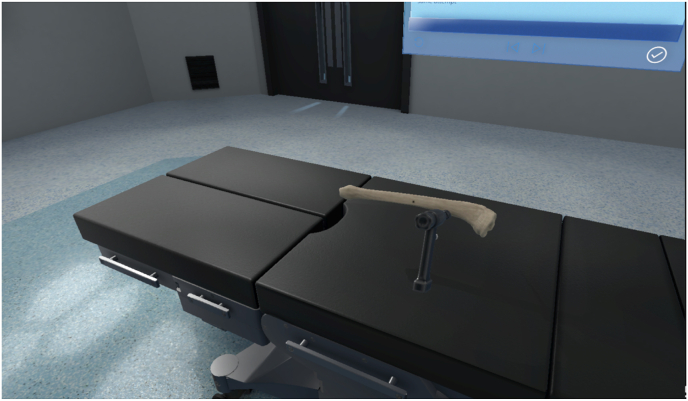
VR Tibia bone as visualised by participants within the FundamentalVR Software.

The VR module consisted of an introductory “sand box” acclimatization space, followed by a training module in which participants were required to drill three holes through a simulated tibia (Fig. 2). The training module instructed participants in the use of a surgical drill, and how to drill through both cortices of a long bone while minimising drill plunge depth beyond the far side of the bone to minimise tissue damage. The haptic group received haptic feedback upon use and manipulation of the drill, including release of resistance on the drill as each cortex was successfully punctured, while the non-haptic group relied on identical (non-haptic) training, with visual and auditory (change in speed of the drill bit as resistance released) feedback alone. Plunge depth distance in millimetres was given on-screen following each of the three attempts.

**Fig. 3:**
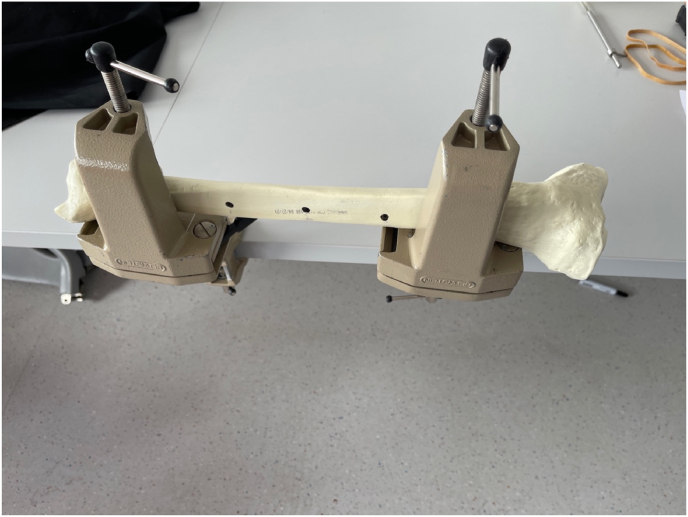
Tibial Saw bone with 2 clamps and 3 marked holes for drill targets.

**Fig. 4:**
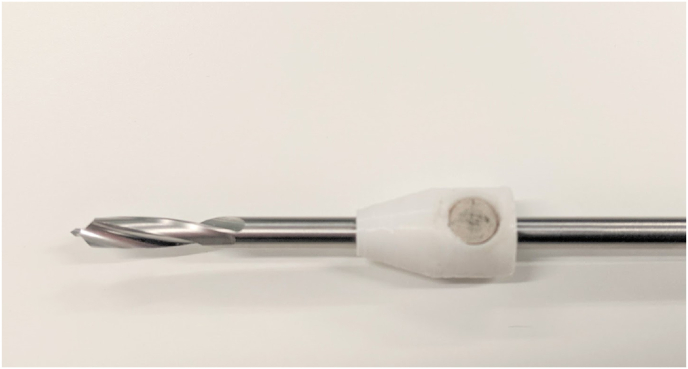
**Custom** plunge Gauge on drill bit. Custom 3D built plunge gauge which moves freely along the drill bit. Small magnet situated within the drill bit to limit unnecessary movement of the plunge gauge as the participant drills through the tibial sawbone.

**Fig. 5:**
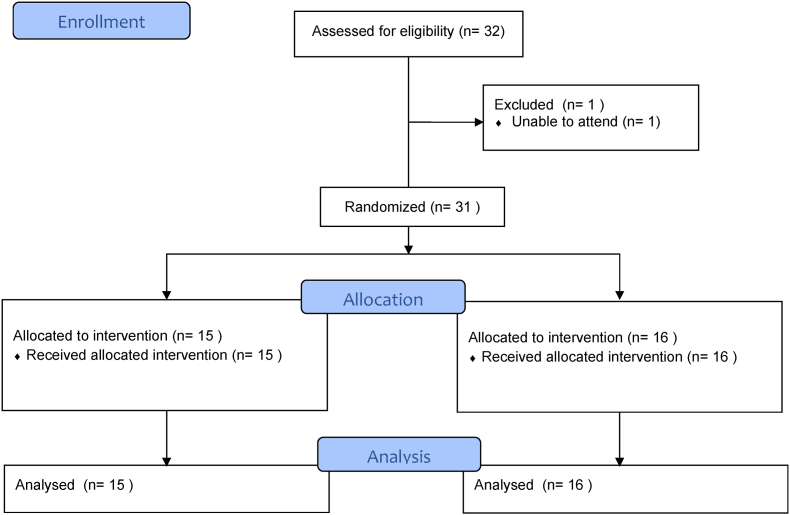
CONSORT flow diagram.

Following completion of the virtual reality training module, participants completed an ex vivo equivalent task by drilling three holes on a benchtop simulated bone (mics, Malmö, Sweden) (Fig. 3). Ex-vivo attempts were video recorded for OSATs assessment and plunge depth for each drill attempt recorded using a custom depth gauge device mounted on the drill bit (Fig. 4). Plunge depth was measured using a custom designed magnetic marker attached to the drill bit to record drill bit plunge depth, from which bone depth as measured by a surgical depth gauge was subtracted. Depths were recorded individually for each drill hole, thereby accounting for differences in bone width depending on location and drill angle (see Fig. 5).

A post-procedure questionnaire was completed by participants to gauge participants' feedback on the study and its use of haptic feedback simulation following which the study was complete for each participant.

### 2.2 Outcomes and analysis

Benchtop drilling performance was compared considering time and plunge depth. The number of participants achieving a clinically safe drill depth (which was adjudged to have been met if < 5 mm plunge depth on at least 2 of 3 attempts) was compared between groups. Learning curves were compared, with a relative plateau considered achieved if participants demonstrated <50% reduction in successive drill attempts, with any increase in plunge depth no more than 2 mm compared to the previous attempt to allow for natural variation.

Video performance of the benchtop drilling were compared and rated by two expert consultant surgeons using the relevant domains of the well-validated Objective Structured Assessment of Technical Skill (OSATS) rating scale. The consultants were selected based on their expertise in orthopaedic surgery and surgical education. Ratings were agreed following piloting and calibration between the two raters. Video raters and researchers were blinded to group allocations during analysis. Pre- and post-procedure questionnaires were also compared between groups.

Non-parametric tests were used to compare between groups. Analysis was performed in Rstudio version 1.4 (Rstudio, Boston, USA). Statistical significance was presumed at p < 0.05.

## 3 Results

### 3.1 Prior knowledge and experience

A total of 31 participants completed the study; 15 were allocated to the haptics group and 16 to the non-haptics group (see Fig. 3). Experience and knowledge prior to simulation-based training was similar between the two groups (Table 1). Fifteen of the participants had no prior experience of orthopaedic drill use and there was large variation in prior operative experience with operative case numbers (assisted or performed) ranging from 0 to 500 procedures. Whilst most participants (84%, 26/31) had some experience with PC or console games, fewer (61%, 19/31) had previously used VR or immersive games and only 6 (19%) had prior experience of orthopaedic simulation platforms.

**Table 1:** Experience/knowledge prior to simulation-based training for haptic and non-haptic groups. Data was gathered through a self-reported questionnaire where participants were asked to rate experience on a scale of 1 (no prior experience/knowledge) to 5 (extensive prior experience/knowledge).

	Haptic	Non-haptic
Operative cases assisted	44 (77)^†^	38 (86)^†^
Operative cases performed	11 (29)^†^	15 (50)^†^
Use of domestic drill	1 (0.5, 3)	1 (0, 2)
Use of drill in operating room	1 (0, 1.5)	0 (0)
Safe surgical use of orthopaedic drill	1 (0, 2.5)	0 (0, 2)
Familiarity of anatomical structures of tibia	2 (2, 3)	2 (1.75, 3.25)
Use of PC or console games	3 (1.5, 4)	2.5 (1, 3)
Experience of VR/Immersive games	1 (0, 2)	1 (0, 2)
Prior use of orthopaedic simulation platform	0 (0)	0 (0)

Data presented as median (IQR) expect ^†^mean (SD).

### 3.2 Bone drilling simulation outcomes

The mean time for drilling completion varied from 7 to 36 s between participants and was significantly shorter in the non-haptics group (13.8 vs 17.5 s, p = 0.027) (Table 2). Mean plunge gap depth, defined as the distance the drill bit was advanced after exit from the cortical bone was significantly reduced in the haptics group (7.6 mm vs 13.6 mm, p = 0.012) (Table 2). A safe drill (<5 mm plunge gap in at least 2 out 3 attempts) was achieved in 40% (6/15) of participants in the haptics group compared with none in the non-haptics group (p = 0.02).

**Table 2:** Simulated clinical outcomes from bone drilling task.

	Haptic	Non-haptic	P
Plunge gap distance (mm)	7.6 (4.3)	13.6 (7.4)	0.012*
Drilling time (sec)	17.5 (4.0)	13.8 (4.2)	0.027*
Safe drill of <5 mm plunge gap in at least 2 out 3 attempts	6 (40)	0 (0)	0.02^¶^*
Learning curve plateau	7 (46.7)	1 (6.3)	0.01^¶^*
OSAT Ratings			
Time/motion	3 (2.5, 4.5)	3 (2, 3)	0.03*
Instrument handling	4 (2.5, 4)	2.5 (2, 3)	0.053
Procedural flow	3 (2,4)	2 (2, 3)	0.029*
Overall performance	4 (2.5, 4)	2.5 (2, 3)	0.051
Combined Scores	14 (10, 17)	8.5 (7.75, 12)	0.0006*

Data presented as mean (SD), absolute number (%) or median (IQR), *<0.05, Mann–Whitney *U* test except ^¶^χ2 test. OSAT *Objective Structured Assessment of Technical Skill*.

A learning curve plateau was achieved in significantly more participants in the haptics group (7/15, 46.7% haptic group vs 1/16, 6.3%, non-haptic group, p = 0.01).

### 3.3 Objective Structured Assessment of Technical Skill (OSATS) ratings

Median combined OSATS ratings (maximum possible score of 20) were significantly improved in the haptics group (14 vs 8.5, p = 0.0006) (Table 2). When analysing individual OSATS rating domains (scored from 1 to 5) median score was significantly higher in the haptics group than the non-haptics group for procedural flow (3 vs 2, p = 0.029) and time (3 vs 3, p = 0.03). Instrument handling and overall performance ratings were not significantly different between the two groups.

### 3.4 Post-simulation participant feedback

Through a self-reported questionnaire participants were asked to rate a series of statements following simulation a scale of 1 (strongly disagree) to 5 (strongly agree) (Table 3). Whilst there were no differences in overall simulation enjoyment and perceived educational value, participants in the haptics group reported the instruments felt and sounded more realistic (median score 4 vs 3, p = 0.006 and median score 5 vs 4, p = 0.03 respectively). Both groups reported that simulation improved both theoretical knowledge and instrument handling (median scores 4). However, the haptics group reported higher ratings for the role of simulation in recognising when to stop drilling (median score 4 vs 3, p = 0.039).

**Table 3:** Post-simulation participant feedback. Data was gathered through a self-reported questionnaire where participants were asked to rate a series of statements on a scale of 1 (strongly disagree) to 5 (strongly agree).

	Haptic	Non-haptic	P
Enjoyed using simulator	5 (5)	5 (4, 5)	0.11
Found valuable	5 (4,5)	4.5 (4, 5)	0.53
Instrument looked realistic	5 (4, 5)	4 (3.75, 5)	0.27
Instrument felt realistic	4 (4, 5)	3 (2, 4)	0.006*
Instrument sounded realistic	5 (4, 5)	4 (3, 5)	0.03*
Bone model appeared realistic	4 (4, 5)	4 (3.75, 5)	0.24
Simulated realistic clinical scenario	4 (4, 5)	4 (3, 5)	0.2
Haptic feedback crucial for task	5 (5)	5 (4.75, 5)	0.68
Would use simulator again if available	5 (5)	5 (4, 5)	0.12
Simulation should be part of surgical training	5 (5)	5 (4, 5)	0.48
Simulation improved theoretical knowledge	4 (3,4.5)	4 (3, 5)	0.85
Simulation trained how to use instrument	4 (4)	4 (3.75, 5)	0.71
Simulation trained how to avoid over-drilling	4 (4)	3 (3, 5)	0.58
Simulation trained how to recognise when to stop drilling	4 (4, 5)	3 (3, 4)	0.039*
Simulation would help improve clinical outcome when operating	4 (4, 5)	4 (3, 4.25)	0.093
Simulation increased confidence in surgical tools	4 (4)	4 (3, 4.25)	0.7
Simulation increased competence in safe use of surgical tools	4 (3.5, 4.5)	3.5 (3, 4.25)	0.32
Regular use of surgical skills training simulation would be valuable	5 (4, 5)	4 (4, 5)	0.53

Data presented as median (IQR), *<0.05, Mann–Whitney *U* test.

Both groups felt that haptic feedback was crucial for completion of the task (median score 5). They both reported they would like to use the simulator again (median score 5) and that it should be part of surgical training (median score 5).

## 4 Discussion

This randomised, controlled, double-blinded study is the first, to our knowledge, to appropriately assess the effect of haptic feedback in virtual reality training, and incorporates cutting edge technology and its effect on clinically relevant outcomes. It strongly suggests that haptic feedback-based VR training is superior compared to non-haptic training. The results of this study suggest an amelioration of learning curve, wherein 46% of the haptic-trained group exhibited outcomes suggestive of a plateau phase, compared with 6% of the non-haptic trained group, and superior outcomes, with a 44% reduction in plunge depth of the drill bit beyond target tissues, a significantly higher percentage of subjects achieving safe drill depths (40% vs none), and better expert-rated performance with higher OSATS scores.

These findings support those of several earlier studies assessing surgical haptics. Strom et al. assessed the effect of haptics in simulated laparoscopic diathermy tasks in a randomised crossover study [23] using the MIST-VR system, a very early laparoscopic trainer with rudimentary software, in 2006. In a total of 36 surgical residents, those randomised to an initial 2-h simulator training sessions with haptic feedback performed significantly better in two diathermy tasks than participants without haptics. Kim et al. compared a standard box trainer (i.e., with real-world haptic feedback) to a virtualised version with basic software, without haptics (2004). They concluded that haptic training resulted in quicker task completion, and greater degree of technical skill improvement [24]. Still other studies, such as Hagelsteen et al. (2017), have demonstrated improved task completion times following haptic training using a more modern platform (LapSim, SurgicalScience, Goteborg, Sweden), though these more recent studies have included lay or student subjects only [25]. Still other studies have reported no differences in outcomes such as accuracy, movement economy and speed of hand movement [26,27].

Most, if not all, of these previous studies have historically suffered from significant limitations. Inappropriate subject groups, sample sizes, training interventions, and testing tasks are in contravention with the current recommendations of the American Psychological Association, the leading body for such research [28]. Our study has sought to overcome these by including only appropriate study candidates in the form of junior trainees and surgeons, clinically relevant tasks and outcomes, and a relatively large sample size.

A further consideration of all simulation-based studies is the validity of the simulation and advancement of technology. Historically, haptic technology and associated software have struggled to approximate real world force feedback, in some situations leading even to haptic feedback being seen as a negative characteristic. Vapenstad et al., for example, evaluated feedback from 20 surgeons who were asked to test VR simulator handles with or without haptic feedback [29]. Whilst most participants felt that handles with realistic haptic feedback were important (85%) in VR simulation, after testing both devices most preferred the handles without haptics (90%). In this study, participants felt that the perceived friction in the haptic handles was too high which negatively impacted the realism of the simulation [29]. Thompson et al., similarly, tested a haptics system which upon assessment was given an expert user rating of only 6/10 for realism of the haptic feedback, suggesting they were testing an underdeveloped haptics system [27]; authors suggested this may have contributed to the lack of any significant differences between assessed groups. In contrast, however, in our study face validity evidence for the system used in this study was very strong, with participants strongly agreeing with the audio, visual, and haptic (in the haptic group) representation of the simulation. Whilst both the haptics and non-haptics group found the simulation model improved their instrument handling, the haptics group reported the instruments felt and sounded more realistic. Crucially, the haptics group reported higher ratings for the role of the simulation in recognising when to stop drilling. This was supported by the reduced plunge gap depth and greater number of participants achieving a safe drill in the haptics group. There are clear clinical implications with these findings, as increased plunge depth risks damage to surrounding tissues. For instance, in femoral fracture plating the superficial femoral artery can be as close as 8 mm from the tip of the plate screw, putting it at significant risk of iatrogenic injury [30].

Overall, this study supports the role of haptic integration in VR simulation models, with simulation technology now at a stage where this can allow the improved acquisition of transferable surgical skills compared to conventional VR simulation. The non-haptic group completed the assessment task more quickly, but with significantly poorer performance, highlighting the difference between quick surgery, and high-quality surgery. The study is strengthened by its double-blinded randomised methodology reducing the risk of selection and observer bias. It also benefits from the use of a OSATS ratings, a well validated tool for assessing surgical skills which allows effective comparison of a range of metrics between the two groups [31].

Further study is required to elucidate the generalisability of this study's findings to other surgical contexts and users. While an ex vivo assessment model was used as opposed to an in vivo assessment of bone drilling, the Sawbones model is a very well validated benchtop model and was felt to represent an accurate simulation for this simple task [32]. While the task used for this study was relatively simple, it was selected to be appropriate for the junior trainees recruited to the study, and one where the haptic feedback required was relatively coarse. With reference to the haptic feedback technology, on the one hand it is yet unclear whether this technology is applicable to finer gradations of haptics, more complex tasks, and more expert users. On the other, however, VR-based simulation has long been espoused as most appropriate for overcoming the initial steep learning curve of the relatively novice learner, for which advanced haptics make not be required. With the development of novel ‘wearable’ haptic feedback technology such as haptic gloves or exoskeletal systems capable of delivering much more advance sensory experiences, on-going research into the impact of these cutting-edge technologies in surgical training is crucial to truly understand their educational value [33].

## 5 Conclusion

This study demonstrates superior performance for a bone drilling task when taught using a VR-based simulation model incorporating haptic feedback, compared to one without haptics, supporting the pursuit and implementation of haptics in surgical training simulation models to improve their educational value. As technology continues to improve and these systems become more cost-effective further research should focus on how these devices can be best utilised in a training environment, facilitating the acquisition of key surgical skills without risking harm to patients.

## Provenance and peer review

Not commissioned, externally peer-reviewed.

## Ethical approval

Local institutional approval – St Georges University NHS Trust.

## Sources of funding

There were no funding sources or sponsors to this study.

## Author contributions

Abrar Gani – Conceptualization, Data Curation, Formal Analysis, Writing – Original Draft, Project Administration.

Oliver pickering – Formal Analysis, Writing – Original Draft.

Caroline Ellis – Data Curation, Formal Analysis.

Omar Sabri – Conceptualization, Methodology, Formal Analysis, Supervision.

Philip Pucher – Conceptualization, Methodology, Formal Analysis Supervision.

## Registration of research studies

Name of the registry: Research Resgistry
Unique Identifying number or registration ID: researchregistry8113
Hyperlink to your specific registration (must be publicly accessible and will be checked): https://www.researchregistry.com/register-now#home/registrationdetails/62d559598d232d001eb902dc

## Consent

Verbal and written consent provided by all participants in the study.

## Guarantor

Abrar Gani, Department of Trauma and Orthopaedics.

St George's University Hospitals NHS Foundation Trust.

Blackshaw Road, Tooting, London, SW17 0QT.

Email: abrar.gani1@nhs.net.

## Declaration of competing interest

2 authors, Omar Sabri and Philip Pucher received consulting fees from Fundamental Surgery for their expertise in their respected surgical speciality. The authors confirm that Fundamental Surgery provided no direct funding for this study, and were not involved in, or oversight of, data collection, analysis, or in the preparation of this manuscript.
